# Level of knowledge, acceptability, and willingness to use oral fluid HIV self-testing among medical students in Kilimanjaro region, Tanzania: a descriptive cross-sectional study

**DOI:** 10.1186/s12981-020-00311-1

**Published:** 2020-09-09

**Authors:** Proches Arobogast Vara, Luyeye Sostenes Buhulula, Fatma Aziz Mohammed, Bernard Njau

**Affiliations:** 1 Kilimanjaro Christian Medical University College, Box 2240, Moshi, Tanzania; 2 Kilimanjaro Christian Medical Centre, Box 3010, Moshi, Tanzania

**Keywords:** Knowledge, Acceptability, Willingness, HIV self-test, Medical students, Tanzania, Sub saharan Africa

## Abstract

**Background:**

HIV Self-Testing (HIVST) is universally accepted as an innovative strategy complimenting existing HIV testing services to archive the UNAIDS, 95-95-95 goals by 2030. However, the adoption of HIVST is lagging in most sub-Saharan countries, including Tanzania. This study aimed to determine the level of knowledge, acceptability, and willingness to use HIVST among Medical students in Kilimanjaro region, Tanzania.

**Methods:**

A descriptive cross-sectional study using a self-administered, semi-structured questionnaire was conducted from May to June 2019 among 271 medical students aged 18–44 years enrolled in a degree of Medicine course at Kilimanjaro Christian Medical University College.

**Results:**

A total of 271 participants were enrolled (response rate of 100%). The mean age was 23.9 (SD 2.9), the majority (91%) were Christians, being single (92%), and a half (50.2%) were males. More than half (55.7%) was sexually active, 67.5% reported the age of first sexual debut at 19 years and above. The majority (81.5%) reported that they had one sexual partner, 37% used condoms during the last sexual act. The majority (98.7%) had never had a sexually transmitted disease during the past 3 months, 22.5% reported using alcohol when having sex. More than three-quarters (79%) ever tested for HIV, and 41.6% tested for HIV in the past year. More than two-thirds (67.9%) had a high level of knowledge on oral fluid HIV self-test. Being a female was related with high level of knowledge (P = 0.225). The acceptability of HIVST was 62.7%, and about two-thirds showed a willingness to buy a self-test kit if available for public use.

**Conclusions:**

The high level of knowledge on oral fluid HIV self-testing, acceptability and willingness to buy self-test kit if available for public use among sexually active medical students underscores the importance of introducing HIVST as a complementary approach for existing HIV testing services in this setting. To make HIVST effective, interventionist should address concerns associated with self-testing, such as lack of pre and post-test counseling, suicidal risks after receiving HIV positive results, perceived risks of inaccurate HIVST test results, lack of linkage to care of individuals receiving HIV positive results, perceived risks of intimate partner violence, coercive testing of a female partner, and perceived high cost of buying self-test kits.

## Introduction

In 2017, it was estimated that 36.9 million [31.1–43.9 million] people were living with HIV, and 21.7 million [19.1–22.6 million] were accessing Antiretroviral therapy (ART) globally. Also, 1.8 million [1.4–2.4 million] became newly infected with HIV while the mortality of people who died from AIDS-related illnesses in 2017 was 940 000 [670 000–1.3 million] worldwide [1].

The UNAIDS has introduced an ambitious global 95-95-95 goals by 2030. The aim is to reach 95% of adults who know their HIV status, 95% of those who are HIV positive are enrolled in ART services, and 95% of those who are in ART have viral suppression by 2030. However, existing conventional HIV testing services (HTS) have failed to achieve universal access to HIV prevention, treatment, and care, especially for non-testers with low access and those at higher risk of HIV acquisition and hard-to-reach populations [1].

In sub-Sahara Africa (SSA), only 40% of people living with HIV (PLHIV) know their HIV status despite a large number of HIV testing service centers [1]. In Tanzania, 52.2% of PLHIV ages 15–64 years know their HIV positive status, with higher proportions among females compared to their male counterparts. Also, only 47% are on ART, and less than 50% are virally suppressed [2]. The low uptake of conventional HTS is associated with stigma and discrimination [3–6], ignorance on the low risk of HIV acquisition, perceived lack of privacy and distance to testing sites especially people in remote areas, long waiting time and lack of confidentially of test results [5, 7–9].

Existing evidence suggests that by allowing people to test discreetly and conveniently, HIVST may increase uptake of HIV testing among people not reached by other HIV testing services [10]. According to the WHO, HIVST is defined as: “a process whereby someone collects his or her specimen (oral fluid or blood) and then performs an HIV test and interprets the result, often in a private setting, either alone or with someone he or she trusts” [1].

Benefits of HIVST reported in the literature, including reaching first-time testers, older men, and very young and vulnerable people, men who sex with men (MSM), commercial sex workers, and transgender [10–15]. Also, HIVST has the advantages of convenience, privacy, anonymity, and a short time to get results, confidentiality, and accessibility. HIVST also has the potential to circumvent barriers related to visiting health facilities or HIV testing points associated with stigma and fear of visibility [10–12].

The WHO has introduced HIVST guidelines recommending HIVST as a potential innovation that could complement the existing HTS to close critical gaps in HIV testing coverage globally [11].

Despite the WHO recommendations, many African countries, including Tanzania are lagging behind the adoption of HIVST in their national HTS policies [16, 17]. The main challenges put forward by HIV policymakers and government stakeholders related to the adoption and implementation of HIVST include perceived risks of inaccurate results, psychological risks because of lack of counseling, and linkage to care of individuals receiving HIV positive results [10, 12, 16, 18–21]. Despite limited evidence of potential psychological risks from HIV self-testing, most arguments against HIVST are mainly based on vague fears [10, 22–25]. However, lack of face-to-face counseling, which is unique for HIVST, remains the most persistent argument against HIVST [10–12, 16, 17, 23, 26–29].

Tanzania’s HIV testing guidelines acknowledge the importance of HIVST and may be considered as the feasible option in the future but currently, HIVST and self-test kits are not permitted for public use in Tanzania, unless for research purposes only [30]. Despite the above-mentioned policy challenge, it is imperative to gather information on the level of knowledge, acceptability, and willingness to use oral-fluid HIV self-testing among medical students in the Kilimanjaro region in Tanzania. Medical students, most of whom fall under the age group of 15–24 years are not exempted from the increased risk of HIV acquisition. A recent Tanzania’s HIV indicator report indicates that HIV prevalence is 5.7% among those under the age group of 15–24 years (national HIV prevalence = 5.5%). However, awareness of HIV-positive status is 50.2% in the same age group [2].

## Materials and methods

### Study setting and study design

This was a cross-sectional study conducted from 1st June to 31st July 2019, at Kilimanjaro Christian Medical University College (KCMUCo) [31]. KCMUCo is a religious health care professional training college situated in the Moshi urban district, which is one of the six districts of the Kilimanjaro region in the North-Eastern part of Tanzania. At the time of this study, KCMUCo has an approximately 1,724 health professional students for the 2018/2019 academic year. Out of those, 1,062 (61.6%) students are from the Faculty of Medicine, and 876 (82.5%) are undertaking a degree in Medicine. The study population included undergraduate medical students aged 18 years and above undertaking a degree in Medicine in year one (MD 1), year three (MD 3), year four (MD 4) and year five (MD 5). During the study period, the KCMUCo had no medical students in year two (MD 2).

The following assumptions were used to arrive at the required minimum sample size. We used Epi-Info statistical software to calculate the sample size. Acceptability of HIVST was estimated based on the study conducted among college students in South Africa (87.1%). To detect a 10% difference in the proportion of acceptability with 80% power a sample of 246 was needed. An additional 10% of the estimated size was added to adjust for drop-out (non-response rate), giving the final sample size of 271.

The primary sample frame was a list of all medical students from MD 1, MD 3, MD 4 & MD 5 forming the sampling units in the study. Within each class, students were categorized by gender (a ratio of 1:1 for males and females), and a systematic sampling frame was employed to select 271 eligible Medical students to participate in the study. Every year level formed a secondary sampling frame. To acquire a truly random sample a list of all medical students in a secondary sampling frame was randomized before determining the sampling frame. To decide on the sampling interval, the study team first calculated the proportion of each year level towards the total population of the undergraduate medical student. The results were used to determine the sample size per class. The sampling interval (nth) was calculated by dividing the number of students in a class (N) by the class sample size (n).

### Data collection procedure and data collectors

The students in each class were organized alphabetically and the first student was selected blindly using a table of random numbers, and the remaining students were selected at a regular interval (nth) from the secondary sampling frame. This process was repeated until the required 271 sample size was achieved. The estimated data collection period was 4 weeks (~ 28 days), however, students are present mostly weekdays (Monday-Friday), which was equal to 20 days. Therefore the total number of students who were recruited per day = total number of 271 divides by 20 was equal to 13.55, ~ 14 students per day, this was equivalent to 266 students for 19 days and 5 students in the final day of data collection. Table 1 presents the sampling procedures of study participants.

Primary data were collected using a pre-tested self-administered, semi-structured questionnaire adopted from South Africa [23], and adapted to elicit a response on demographic variables, sexual risk behaviors, and level of knowledge regarding HIVST, HIV testing practices, acceptability, and willingness to use oral fluid HIVST. Acceptability and willingness to use oral fluid HIVST were the dependent variables in this study. Demographic variables include sex, age, religion, year of study, and tribe.

**Table 1:** Demographic, sexual and testing practices of medical students (N = 271)

Variables	Total	Males	Females
	N= 271 (%)	n= 135 (49.8%)	n= 136 (50.2%)
Age group			
18–24 years	187 (69.0)	84 (62.2)	103 (75.7)
25 and above	84 (31.0)	51 (37.8)	33 (24.3)
Marital status			
Single	251 (92)	124 (91.8)	127 (93.4)
Married	7 (2.6)	4 (3.0)	3 (2.2)
Cohabiting	13 (4.8)	7 (5.2)	6 (4.4)
Religion			
Christian	239 (88.2)	123 (91.1)	116 (85.3)
Muslim	30 (11.1)	12 (8.9)	18 (13.2)
Others	2 (0.7)	0 (0.0)	2 (1.5)
Year of study			
First year	62 (22.9)	31 (23.0)	31 (23.0)
Third year	74 (27.3)	37 (27.4)	37 (27.2)
Fourth year	81 (29.9)	41 (30.4)	40 (29.4)
Fifth year	54 (19.9)	26 (19.2)	26 (20.4)
Ever had sex			
Yes	151 (55.7)	94 (69.6)	57 (41.9)
No	120 (44.3)	41 (30.4)	79 (58.1)
Age of 1st sexual debut	(n = 151)	(n = 94)	(n = 57)
Below 15 years	9 (6.0)	9 (9.6)	0 (0.0)
15–18 years	40 (26.5)	36 (38.3)	4 (7.0)
19 years and above	102 (67.5)	49 (52.1)	53 (93.0)
Number of sexual partners in the past year	(n = 151)	(n = 94)	(n = 57)
One partner	123 (81.5)	67 (71.3)	56 (98.2)
Two partners	17 (11.3)	16 (17.0)	1 (1.2)
More than two partners	11 (7.2)	11 (11.7)	0 (0.0)
Condom use during the last sexual act	(n = 151)	(n = 94)	(n = 57)
Yes	56 (37.0)	38 (40.4)	18 (31.6)
No	95 (63.0)	56 (59.6)	39 (68.4)
Ever had sexual transmitted disease during the past 3 months	(n = 151)	(n = 94)	(n = 57)
Yes	2 (1.3)	1 (1.1)	1 (1.8)
No	149 (98.7)	93 (98.9)	56 (98.2)
Used alcohol when having sex	(n = 151)	(n = 94)	(n = 57)
Yes	34 (22.5)	19 (20.2)	15 (26.3)
No	117 (77.5)	75 (79.8)	42 (73.7)
Ever tested for HIV	(n = 271)	(n = 135)	(n = 136)
Yes	214 (79.0)	109 (80.7)	105 (77.2)
No	57 (21.0)	26 (19.3)	31 (22.8)
Most recent HIV test	(n = 214)	(n = 109)	(n = 105)
Less than a month	21 (9.5)	13 (12.0)	8 (7.4)
1-2 months	24 (11.2)	13 (12.0)	11 (10.5)
3 months or more	82 (38.2)	43 (39.3)	39 (37.1)
One year or more	87 (41.6)	40 (36.7)	47 (45.0)

### Level of knowledge on oral fluid HIVST

Ten questions were used to assess the level of knowledge on oral fluid HIVST (e.g., “What is the appropriate time to read the oral fluid HIV test?”). The expected response categories ranged from 1 = “Immediately” to 6 = “I don’t know.”

For each correct response was scored 1 point and incorrect and nonresponse was scored 0 points. The overall score ranged from a minimum of 0 to a maximum of 10. Those who had a mean score of 5 points and above were categorized as having a high level of knowledge and those who had a mean score of below 5 were categorized as having a low level of knowledge on oral fluid HIVST. Participants viewed a professionally developed a 5-minute video to introduce the concept of oral-fluid HIVST before data collection.

### Acceptability of HIVST

Participants were asked whether they would use an oral-fluid self-test kit should it be available to the general public. The expected response was 1 = Yes, or 2 = No.

### Willingness to use oral fluid HIVST

Three (3) questions were used to assess the willingness to use oral fluid HIVST (e.g., “Are you willing to pay money to buy a self-test kit should it be available to the general public?”). The expected response was 1 = Yes or 2 = No.

### Data management and analysis

Two data entry clerks did the double entry of data to ensure the validation of data. Data were cross-cheeked for entry error and range checks before data analysis. The analysis was performed using SPPS version 20 for windows. Frequencies and percentages were used to present categorical variables. To compare the independent variable with continuous dependent variables, the T-test was used of which a p-value of < 5% was considered as a cut-off point to test statistical significance.

## Results

A total of 271 participants were included in the study with a response rate of 100%. More than two-third (n = 187, 69%) was aged between 15 and 24 years with the mean age of 23.9 (SD 2.9). The majority, 91.8% were Christian, 50.2% were male, the majority were single (92%) and 100% were Tanzanian, most of them were obtained from the fourth year (29.9%) and the least were obtained from the fifth year (19.9). More than half (55.7%) was sexually active, 67.5% reported the age of first sexual debut at 19 years and above. The majority (81.5%) reported that they had one sexual partner, and 37% used condoms during the last sexual act.

The majority (98.7%) had never had a sexually transmitted disease during the past 3 months, 22.5% reported using alcohol when having sex. More than three-quarters (79%) ever tested for HIV, and 41.6% tested for HIV in the past year. More than two-thirds (67.9%) had a high level of knowledge on oral fluid HIV self-test.

### Level of knowledge of HIVST

**Fig. 1:**
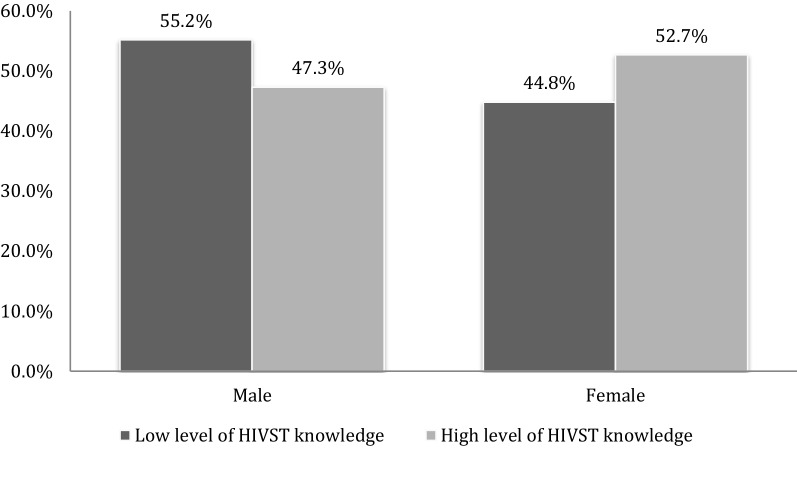
Level of knowledge on HIVST oral fluid self-test by gender (N = 271)

One hundred and eighty-four participants (67.9%) had a high level of knowledge of oral-fluid self-testing compared to 32.1% (n = 87/271) of participants with a low level of knowledge. Female participants had higher proportions of having a high level of knowledge compared to their male counterparts (71.3 vs. 64.4%; P = 0.225). Figure 1 below presents the level of knowledge of study participants by gender.

### Acceptability regarding HIVST

**Fig. 2:**
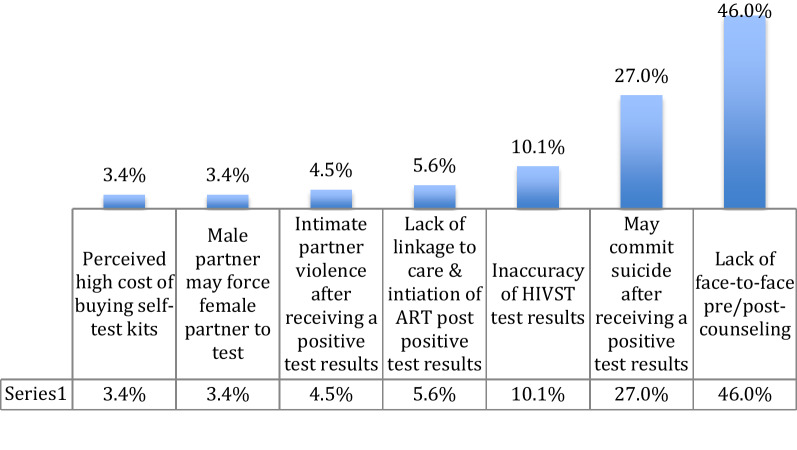
Reasons for HIVST non-uptake of oral fluid self-test (N = 89)

The acceptability of oral-fluid HIVST as a testing option was 62.7% (n = 170/271) among study participants. Also, more than two-thirds (65.3%) of study participants were comfortable in using oral-fluid HIVST, 61.2% were comfortable in using with boyfriend/girlfriend, and 66.4% would recommend the oral-fluid test to their parents, friends, and relatives (data not shown). Two-thirds (62.7%; n = 170/271) of study participants agreed that oral-fluid HIVST should be available for public use. However, 32.8% (n = 89/271) of participants had concerns regarding non-uptake of HIVST. Participants cited the reasons like lack of pre and post-test counseling after self-testing (46%), may commit suicide after receiving a positive HIV test result (27%), inaccuracy of HIVST test results (10.1%), intimate partner violence (4.5%), or coercion to test for HIV using HIVST (3.4%), lack of linkage to HIV care and initiation of ART post-HIVST (5.6%), and perceived high cost of buying the self-test kits (3.4%). Reasons for HIVST non-uptake among study participants are presented in Fig. 2 below.

### Willingness regarding HIVST

Out of 170 participants who reported acceptability of oral-fluid HIVST, 46.5% (n = 79/170) were willing to buy the self-test kits if they were available. The majority, 96.2% (n = 76/79) were willing to pay Tanzanian shillings (TZS) 1,000 to 3,000 (1 USD = 2310 TZS), to buy the self- test kits.

### Factors associated with acceptability of oral-fluid HIVST

The primary outcome of this study was the acceptability of oral-fluid HIVST. In a bivariate analysis as depicted in Table 2, five factors were significantly associated with the acceptability of HIVST among medical students. The five factors that were significant at p < 0.05 levels were: age group, being sexually experienced, age of sexual debut, number of lifetime sexual partners, and condom use during the last sexual act. Participants who were aged 25 years and above were less likely to report acceptability of HIVST (Odds Ratio (OR = 0.54; 95% CI 0.31–0.93; P = 0.03). Acceptability of HIVST was associated with being sexually experienced (OR = 2.77 [95% CI 1.67–4.59] P = 0.000), age of sexual debut (OR = 0.38 [95% CI 0.18–0.79] P = 0.01), number of lifetime sexual partners (OR = 2.56 [95% CI 1.04–6. 33] P = 0.04), and condom use during the last sexual act (OR = 0.41 [95% CI 0.19–0.79] P = 0.02). Gender, ever tested for HIV, knowledge of oral-fluid HIVST, and use of alcohol when having sex was not associated with the acceptability of HIVST.

**Table 2:** Factors associated with acceptability of oral-fluid self-testing in bivariate analysis (n = 271)

Variables	Acceptability of HIVST		Chi-square	OR [95% CI]	P value
	Yes (n = 167)	No (n = 104)			
Gender					
Male	76 (45.5)	59 (56.7)	X^2^ = 3.22	1	
Female	91 (54.5)	45 (43.3)		0.64 (0.38–1.0)	0.072
Age group					
18–24 years	107 (64.1)	80 (76.9)	X^2^ = 4.95	1	
25 and above	60 (35.9)	24 (23.1)		0.54 (0.31–0.93	0.03*
Ever had sex					
No	58 (34.7)	62 (59.6)	X^2^ = 16.1	1	
Yes	109 (65.3)	42 (40.4)		2.77 (1.67–4.59)	0.000*
Ever tested for HIV					
Yes	136 (81.9)	78 (75)	X^2^ = 1.87	1	
No	31 (18.1)	26 (25)		1.51 (0.83–2.74)	0.172
Knowledge of oral-fluid HIVST					
Low knowledge	56 (33.5)	31 (29.8)	X^2^ = 0.41	1	
High knowledge	111 (66.5)	73 (70.2)		1.19 (0.70–2.02)	0.52
Age of sexual debut (n = 151)					
Below 18 years	29 (26.4)	20 (48.8)	X^2^ = 6.85	1	
19 years and above	81 (73.6)	21 (51.2)		0.38 (0.18–0.79)	0.01*
Number of sexual partners (n = 151)					
One partner	38 (34.5)	7 (17.1)	X^2^ = 4.36	1	
Two partners or more	72 (65.5)	34 (82.9)		2.56 (1.04–6.33)	0.04*
Condom use during the last sexual act (n = 151)					
Yes	34 (31.2)	22 (52.4)	X^2^ = 5.83	1	
No	75 (68.8)	20 (47.6)		0.41 (0.19–0.85)	0.02*
Used alcohol when having sex (n = 151)					
Yes	24 (21.8)	11 (26.8)	X^2^ = 0.42	1	
No	86 (78.2)	30 (73.2)		0.76 (0.33–1.74)	0.52

*Significant p-value

In the multivariate logistic regression analysis, all five factors were included to confirm the observed association at bivariate analysis. As depicted in Table 3, two variables namely: age of sexual debut and condom use during the last sexual act were found to influence whether a participant reports acceptability of oral-fluid HIVST. The age of participants ever had sex, and the number of lifetime sexual partners did not contribute significantly to the model. Participants who reported had sexual debut below 18 years were less likely to report acceptability of oral-fluid HIVST compared to those who had sexual debut 19 years or older [Adjusted odds ratio (AOR) = 0.37; 95% CI 0.16–0.81]. Acceptability of oral-fluid HIVST was condom use during the last sexual act. Participants who reported non-condom use in their last sexual act were twice as likely to accept HIVST, in comparison to those who had used condoms (AOR = 2.35; 95% CI 1.09–5.06).

**Table 3:** Factors associated with acceptability of oral-fluid HIVST

Variables	Acceptability of HIVST		P value	Unadjusted OR [95% CI]	P value	Adjusted OR [95% CI]^a^
	Yes (n = 167)	No (n = 104)				
Age group						
18–24 years	107 (64.1)	80 (76.9)	0.03*	1	0.37	1
25 and above	60 (35.9)	24 (23.1)		0.54 (0.31–0.93)		0.69 (0.32–1.53)
Ever had sex						
No	58 (34.7)	62 (59.6)	0.000*	1	0.74	1
Yes	109 (65.3)	42 (40.4)		2.77 (1.67–4.59)		1.54 (0.75–3.04)
Age of sexual debut (n = 151)						
Below 18 years	29 (26.4)	20 (48.8)	0.01*	1	0.01*	1
19 years and above	81 (73.6)	21 (51.2)		0.38 (0.18–0.79)		0.37 (0.16–0.81)
Number of sexual partners (n = 151)						
One partner	38 (34.5)	7 (17.1)	0.04*	1	0.54	1
Two partners or more	72 (65.5)	34 (82.9)		2.56 (1.04–6.33)		1.33 (0.54–3.25)
Condom use during the last sexual act (n = 151)						
Yes	34 (31.2)	22 (52.4)	0.02*	1	0.03*	1
No	75 (68.8)	20 (47.6)		0.41 (0.19–0.85)		2.35 (1.09–5.06)

*Significant P value

^a^Log likelihood test: 163.0; Cox & Snell R^2^ = 0.82; Nagelkerke R^2^ = 0.119

## Discussion

This study aimed to determine the level of knowledge on oral-fluid HIVST, acceptability, and willingness to use oral-fluid HIVST among medical students at KCMUCo in Kilimanjaro region in a period when Tanzania was in a process to adopt HIVST as a complimentary HTS.

The majority of medical students in this study were relatively young and being single. Also, the study observed that more than half of the participants were sexually experienced. Male participants reported a higher proportion of being sexually experienced compared to their female counterparts. This could be explained by the fact that males were more likely to be explicit about their sexual experiences compared to females because of expected social norms on sexuality. This observation corroborates similar findings from studies in other settings [32–36]. Almost two-thirds of sexually experienced participants had a sexual debut after 18 years old. This observation is contrary to a similar study done in the same setting in 2009 reporting early sexual debut with a mean age of 11 years [37], and findings among youths in different settings reporting early sexual debut [32–36, 38–41].

Even though the majority of sexually experienced participants reported having one sexual partner, two-third reported non-condom use in their last sexual act. Given the fact that most participants in this study are unmarried and reporting unprotected sexual intercourse, increasing their vulnerability to HIV acquisition [42].

The uptake of available HTS in the current study is relatively high compared to a previous study conducted in 2009 in the same settings [37] and of students in colleges and universities in other settings [23, 43–45].

The current finding should be attributed to the fact that, since 2017, KCMUco has implemented an HIV & AIDS policy aimed to address discrimination, create awareness and facilitate uptake of HTS, and integrate HIV & AIDS issues into the teaching curriculum and community-based interventions [31].

Since HIVST is regarded as a “new technology”, the study also assessed the students’ level of knowledge on oral-fluid HIVST. More than two-thirds of participants had a high-level knowledge score on HIV oral fluid tests, although most were unaware of HIVST before the survey. Before data collection, all eligible participants viewed an instructional video on oral-fluid HIVST and received instruction sheets on how to use oral fluid self-test kits, which could explain the observed high level of knowledge on HIVST in this setting. However, the gender differences in HIVST knowledge observed in the current study, underscore the importance of having gender-specific interventions to create awareness and basic understanding in the use of HIVST [23].

Two-third of medical students in this study considered oral-fluid to be acceptable if available for public use. Further, the age of sexual debut and condom use during the last sexual act were factors significantly associated with the acceptability of HIVST among the study population. These findings add to the growing evidence demonstrating the acceptability of oral-fluid HIVST in different populations [10, 46–48]. At the time of conducting this study, HIVST in Tanzania was still under consideration for public use. However, in November 2019, the Tanzanian Parliament passed an HIV and AIDS (Prevention and Control) Act, (CAP.431) allowing HIVST for public use [49]. High HIVST acceptability has also been reported with different population groups in a different setting, including Sub-Saharan countries [10, 42, 50–59].

Despite the high acceptability of HIVST reported in the current study, some students had concerns regarding HIVST. These concerns are similar to findings from HIVST studies conducted from different settings. For example lack of pre and post-test counseling after self-testing was a key concern among medical students in the current study. This concern concurs with findings reported from different settings, underlining the importance of face-to-face counseling and HIVST [10, 12, 16, 17, 29, 53, 59]. Pai et al. [57], however, reported different opinions regarding counseling, ranging from anonymity, either anonymity or face-to-face counseling, to face-to-face counseling alone.

The cost of buying a self-test kit is one of the major barriers identified in the literature that may hinder adoption, willingness to use, purchase and the uptake of HIVST [10, 12, 53, 60, 61].

Despite medical students reporting their willingness to purchase the self-test kits, more than half were unwilling to purchase the self-test kits. This observation is contrary to findings from studies assessing willingness to HIVST from other settings, which reported a high willingness to purchase self-test kits [23, 62–64].

Further, students who reported willingness to self-test kits were willing to pay TZS. 1,000 to 3,000 (0.42–1.25$). This finding is contrary to medical students in Canada who were willing to pay 20$ (48,000 TZS) to purchase the self-test kit [64]. The most probable explanation to the observed differential on willingness to purchase self-test kits is because of economical difference between Tanzania and Canada. This observation underscores the importance of addressing the cost involved in the acquisition of self-test kits, particularly among people in low-income countries, to increase the uptake of HIVST [10, 20, 61].

Several study limitations need to be noted. The study was a cross-sectional design, which is inadequate for measuring the directionality of the causality of the observed associations. This study enrolled only medical students pursuing a doctor of medicine; hence there is a level of selection bias and the finding of this study cannot be generalized to all students at KCMUCo, or young people in Tanzania. The study asked respondents to recall events that had happened in the past. Recall bias is possible particularly among older participants to remember the exact timing of their first sexual acts and condom use.

Reporting bias could not be excluded from this study because the study relied on self-report responses. Also, there is a possibility of having under-reporting or over-reporting of sexual related practices because of social desirability bias among study participants.

## Conclusion

In conclusion, the high level of knowledge on oral fluid HIV self-testing, acceptability and willingness to buy self-test kit if available for public use among sexually active medical students underscores the importance of introducing HIVST as a complementary approach to facilitate HIV testing in this setting. To make HIVST effective, interventionist should address concerns associated with self-testing, such as lack of pre and post-test counseling, suicidal risks after receiving HIV positive results, perceived risks of inaccurate HIVST test results, lack of linkage to care of individuals receiving HIV positive results, perceived risks of intimate partner violence, coercive testing of a female partner, and perceived high cost of buying self-test kits [10–18, 20]. 

## Data Availability

The datasets used and/or analyzed during this study is not publicly available, but may be available from the corresponding author upon reasonable request, and with permission from Kilimanjaro Christian Medical College.

## Abbreviations

ART: Antiretroviral therapy
HIVST: HIV self-testing
HTS: HIV testing services
PLHIV: People living with HIV
SSA: Sub Sahara Africa
